# Human Bone Typing Using Quantitative Cone-Beam Computed Tomography

**DOI:** 10.1016/j.identj.2022.08.011

**Published:** 2022-09-29

**Authors:** Hairong Huang, Dong Chen, Kurt Lippuner, Ernst B. Hunziker

**Affiliations:** a Department of Osteoporosis, Inselspital Bern University Hospital, Bern, Switzerland; b Hubei-MOST KLOS & KLOBM, School and Hospital of Stomatology, Wuhan University, Wuhan, China; c Department of Orthopaedic Surgery, Inselspital Bern University Hospital, Bern, Switzerland

**Keywords:** CBCT, Bone types, Dental implantology, Human, Quantification

## Abstract

**Introduction:**

Bone typing is crucial to enable the choice of a suitable implant, the surgical technique, and the evaluation of the clinical outcome. Currently, bone typing is assessed subjectively by the surgeon.

**Objective:**

The aim of this study is to establish an automatic quantification method to determine local bone types by the use of cone-beam computed tomography (CBCT) for an observer-independent approach.

**Methods:**

Six adult human cadaver skulls were used. The 4 generally used bone types in dental implantology and orthodontics were identified, and specific Hounsfield unit (HU) ranges (grey-scale values) were assigned to each bone type for identification by quantitative CBCT (qCBCT). The selected scanned planes were labelled by nonradiolucent markers for reidentification in the backup/cross-check evaluation methods. The selected planes were then physically removed as thick bone tissue sections for in vitro correlation measurements by qCBCT, quantitative micro–computed tomography (micro-CT), and quantitative histomorphometry.

**Results:**

Correlation analyses between the different bone tissue quantification methods to identify bone types based on numerical ranges of HU values revealed that the Pearson correlation coefficient of qCBCT with micro-CT and quantitative histomorphometry was *R* = 0.9 (*P* = .001) for all 4 bone types .

**Conclusions:**

We found that  qCBCT can reproducibly and objectively assess human bone types at implant sites.

## Introduction

During recent decades, dental implants have become a widely used treatment option in oral surgery. They serve in a variety of functions: as mechanical support for different types of dental prostheses and as carriers of crowns, bridges, dentures, or orthodontic apparatuses, and so on. In this context, implant stability, generally quantified by noninvasive implant stability quotient measurements (resonance frequency analysis), is often used as a predictive parameter to assess the outcome of an implant placement, and it has proven itself to be quite a useful estimator for the rough clinical estimate of the degree of treatment success that can be expected.^1^ Therefore, it is an indirect assessment of the degree of risk for possible clinical failure. However, clinical key decisions for successful outcomes are already made at the pre-implant planning stage. In the early planning phase when oral surgeons start thinking about placing an implant, they assess the local quality of bone available at the prospective site of implant placement in order to evaluate what degree of risk for failure is to be expected.^2^ For this assessment, surgeons estimate (since the 1980s) on a subjective basis the quality of the local bone and define a bone type that they will have to deal with during surgery. Various subjective categorisation schemes have been suggested to achieve this goal.^3^, ^4^, ^5^, ^6^, ^7^ The most frequently used classification method in clinical practice is based on the radiographic appearance and the resistance to drilling. This was developed by Zarb and Lekholm,^8^ in which essentially 4 different types of bone quality were categorised:

Type 1: predominantly cortical bone
Type 2: a thick layer of cortical bone with a central core of dense trabecular bone
Type 3: a thin layer of cortical bone with dense trabecular bone
Type 4: a very thin layer of cortical bone with low density of trabecular bone

This is a subjective classification scheme, but it is still used today. An observer-independent, objective, and quantitative method with a potential for automation has not thus far been established. This is surprising since radiographic evaluations, and in particular the use of cone-beam computed tomography (CBCT), have undergone considerable technological evolution since the mid-1980s. These technologies were developed to a degree of sophistication and accuracy that theoretically allows an objective, reproducible, and observer-independent quantitative assessment of human facial bone type.^3^^,^^9^, ^10^, ^11^ However, some attempts were made to place selected presurgical evaluation methods on an objective basis, by using different imaging approaches such as routine radiography, tomography, digital panoramic radiography, and resonance frequency analysis.^5^^,^^12^^,^^13^ Moreover, the technical equipment required in order to provide such desired objective information is nowadays generally available in all dental practices and clinics. However, quantitative CBCT (qCBCT) is not a straightforward process; rather, it is associated with a number of pitfalls and artefacts that need to be taken into account. These have been well illustrated both in scientific investigations^14^ and in a comprehensive retrospective clinical study.^15^

It was the aim of this study to establish a simple, objective, and reproducible quantitative (numerical) CBCT-based method to define the 4 basic bone types in the human maxillae and mandibulae in-vitro and to apply adequate validations for cross-checks and backups of the generated CBCT data.

## Methods

### Experimental design

**Fig. 1:**
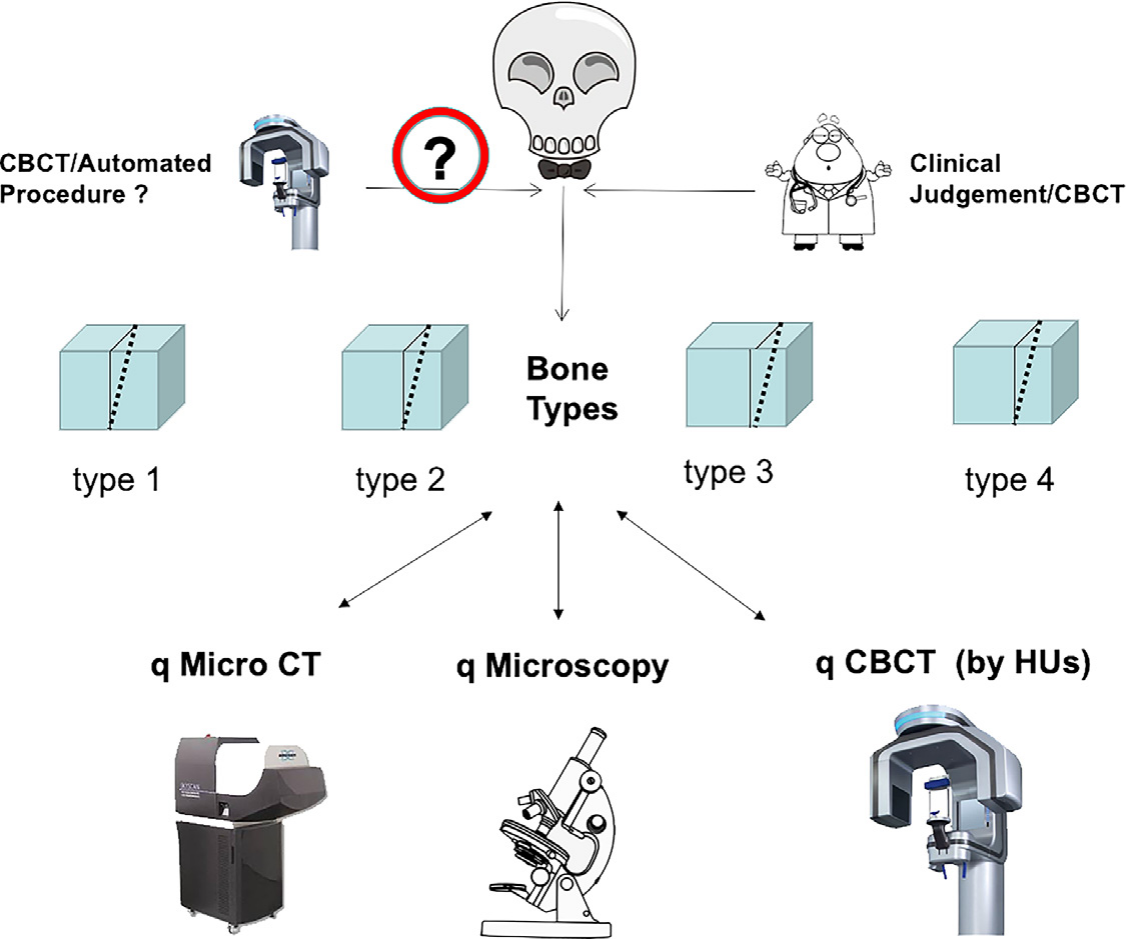
Graphical abstract of the experimental design.

The required facial bones for this in vitro study were obtained from human skulls donated for instructional and experimental use. For validation purposes of the qCBCT approach for bone typing, quantitative micro–computed tomography (q micro-CT) and quantitative histomorphometry of the exact same sites were used in order to establish the reproducibility and objectivity of the qCBCT approach for routine use in clinical practice (see Figure 1 for a graphical abstract of the experimental design).

### Cadaver bone preparation and CBCT scan

Six adult human cadaver skulls (age range, 54 to 78 years; all male in order to avoid any possible sex-related differences, such as may occur in osteoporosis disease) were obtained from the cadaver pool of the Anatomy Department of the School of Stomatology of Wuhan University. Cadavers of this pool are sourced from individual personal donations by written final will documents for use in education and research. To eliminate donors who may have had any potential of a bone-related pathology, the medical history of the cadavers was checked for any general metabolic or bone-related diseases, chemotherapy, long-term analgesic treatment, and so on. These were excluded from the study if found positive in any of these respects.

### Selection of specific bone type areas

Systematic CBCT scans (Planmeca Promax 3D, Planmeca Oy) were made through the maxillae and mandibulae. On these scans, areas of the 4 bone types outlined by Zarb and Lekholm were categorised by 2 independent experienced dental implantologists. The scheme used for the bone typing was the one of Zarb et al.^8^ Each area identified with a specific bone type was then marked with gutta-percha markers (gp markers) at 3 points in order to define the precise location and orientation/direction of that plane in space.

### Calibration of CBCT signals by systematic radiographs of defined increasing concentrations of K_2_HPO_4_ in solution

In order to calibrate the qCBCT signals on an objective and reproducible basis, well-defined increasing concentrations of K_2_HPO_4_ (dipotassium hydrogen phosphate) solutions were used, as previously described^15^: 50 mg/mL, 100 mg/mL, 200 mg/mL, 400 mg/mL, 600 mg/mL, 800 mg/mL, and 1000 mg/mL to be measured by CBCT (Planmeca Promax 3D, Planmeca Oy) and to reconfirm the relationship between grey-scale values and Hounsfield unit (HU) values (a recognised method that had been previously established^15^).

### Quantitative CBCT analysis

The samples were then CBCT-scanned again through these gp-marked planes. For each bone type, the gp-marked planes were remeasured using the Simplant software (Densply Sirona); each sample was quantified 3 times. We defined for each bone type specific ranges of HU values (grey-scale values in the fields of interest) for the numerical quantification of the data for specific correlation to each bone type: type 1 = 2000 to 800; type 2 = 800 to −200; type 3 = −200 to −500; and type 4 = −500 to −900. The mean values of the 3 measurements were then used for further comparative analyses. The appropriate instrument correction factors could then be established.

### Quantitative micro-CT analysis

**Fig. 2:**
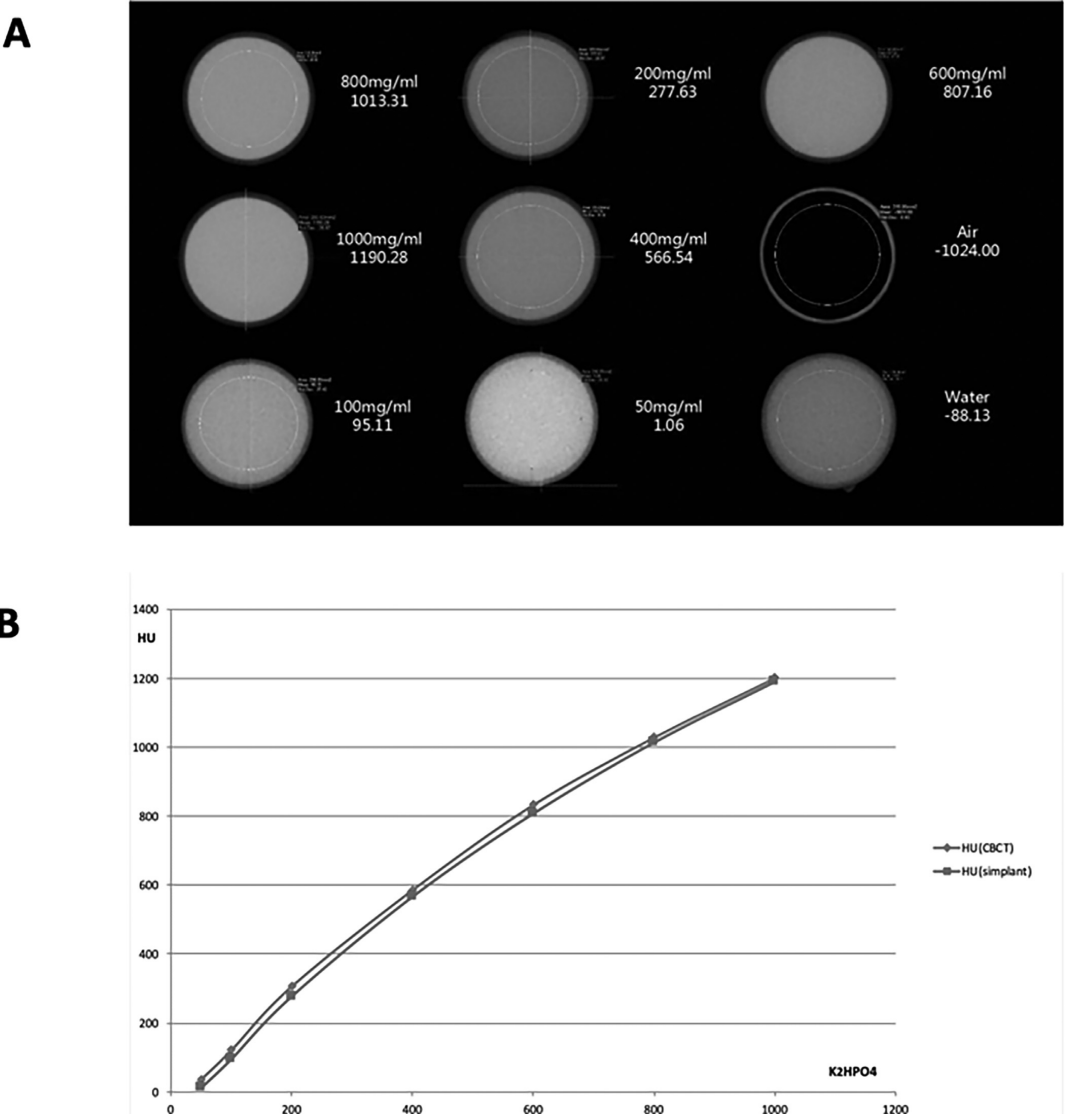
Cone-beam computed tomography (CBCT) instrument calibration by K_2_HPO_4_ and data analysis by 2 different software products. **A**, By using a defined concentration series of K_2_HPO_4_ (dipotassium hydrogen phosphate) electrolyte solution calibration was attained. The solutions were used in the range of 50 mg/mL to 1000 mg/mL K_2_HPO_4_, yielding corresponding Hounsfield unit (HU) values; control reference values were obtained using air and water. The representative CBCT images of these radiographs illustrate the corresponding grey value levels. **B**, CBCT analysis of the calibration solutions of K_2_HPO_4_ was performed by using 2 different software products: blue line = Planmeca Promax 3D (Planmeca Oy) and red line = Simplant 16 (Densply). These 2 analytical tools, based on product specific reconstruction algorithms, delivered identical results, thus providing a basis for data comparison irrespective of the equipment used.

We labelled each plane analysed by qCBCT with 3 small metal tags (at exactly the same location of the gp markers) for precise relocalisation of the scanned planes in each cadaver bone by q micro-CT. The samples were measured in a micro-CT machine (Bruker Skyscan 1172, Bruker) and the technical setup defined as follows: image pixel size: 10 µm; filter size: 1 mm Al; rotation step (deg): 0.4; use of 180 rotation; source voltage: 70 kV; and source current: 100 uA. The thickness of sections/planes was set at 10 μm. Precise remeasuring of the identical plane as measured by qCBCT was thus assured (Figure 1). The calibration of the CBCT measurements in HUs was performed by using a predefined concentration series of K_2_HPO_4_ (see above for details) as well as a negative control (air) and a positive control (water) for optimal adjustment of the device (Figure 2A). The data of the CBCT measurements of the calibration solutions were then analysed by 2 different (commercially available) and frequently used software packages, that is, by Planmeca ProMax 3D (Planmeca Oy) and by Simplant 16 (Densply Sirona), in order to test whether the product-specific reconstruction algorithms yielded different results. Quantitative micro-CT data for bone masses were obtained by using standard software for micro-CT quantification.^16^^,^^17^

### Quantitative histomorphometry

In order to histomorphometrically quantify the bone tissue masses in exactly the same planes as CBCT and q micro-CT had, the gp markers were re-placed at exactly the same location by metal markers (Ag-Hg alloy) that show resistance to the various chemicals used during chemical fixation of the bone tissue, dehydration, and plastic embedding.^18^ Saw cuts of approximately 100 μm thickness through the labelled planes were produced on a Leco VC-50 sawing machine. Sections were then glued to plastic support plates, milled, and polished down to a thickness of 80 µm and surface-stained with McNeil's tetrachrome and toluidine blue O.^18^ The sections were photographed at a final magnification of 40× in a Nikon Eclipse 50i light microscope by using a subsampling scheme according to a systematic random-sampling protocol.^19^ Using the photographic prints, the bone densities were quantified by point counting methods, according to unbiased stereologic principles.^20^

### Statistical analysis

Statistics software (SPSS 21.0, IBM) was used to analyse the data. The correlative relationships between the qCBCT, q micro-CT, and quantitative histomorphometric data was computed by using test statistics for the Pearson correlation coefficient *R*.^21^ Data spread and variability were computed using the coefficient of determination *R*^2^ as a powerful indicator for the goodness of curve fit.^22^ Comparison of data amongst the 3 groups of measurements for statistical differences was performed by applying paired *t* tests.

## Results

**Fig. 3:**
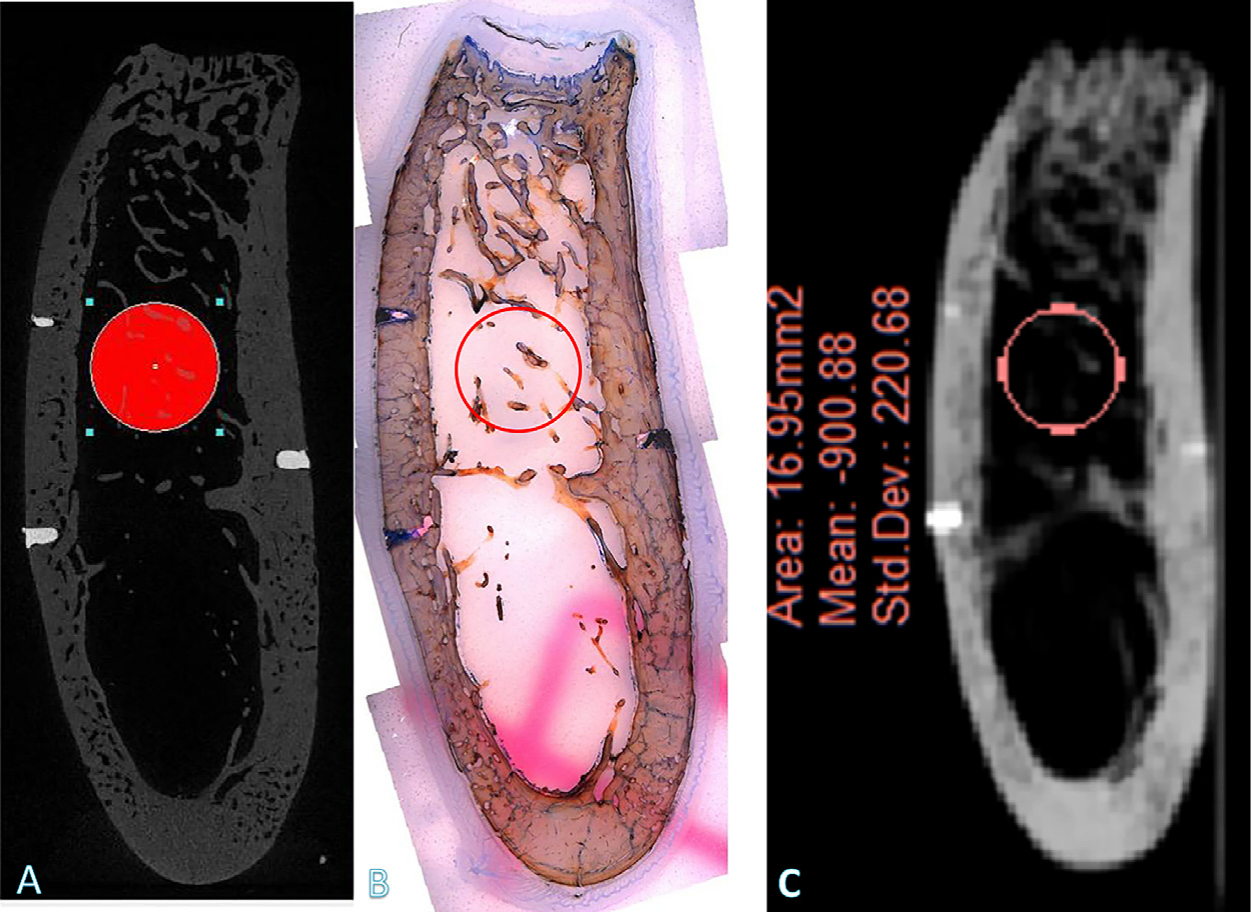
Illustration of the quantitative bone-type measurements. Bone-type measurements were obtained by (A) cone-beam computed tomography (CBCT), (B) histomorphometry, and (C) micro–computed tomography (CT) (representative sample specimen).

**Fig. 4:**
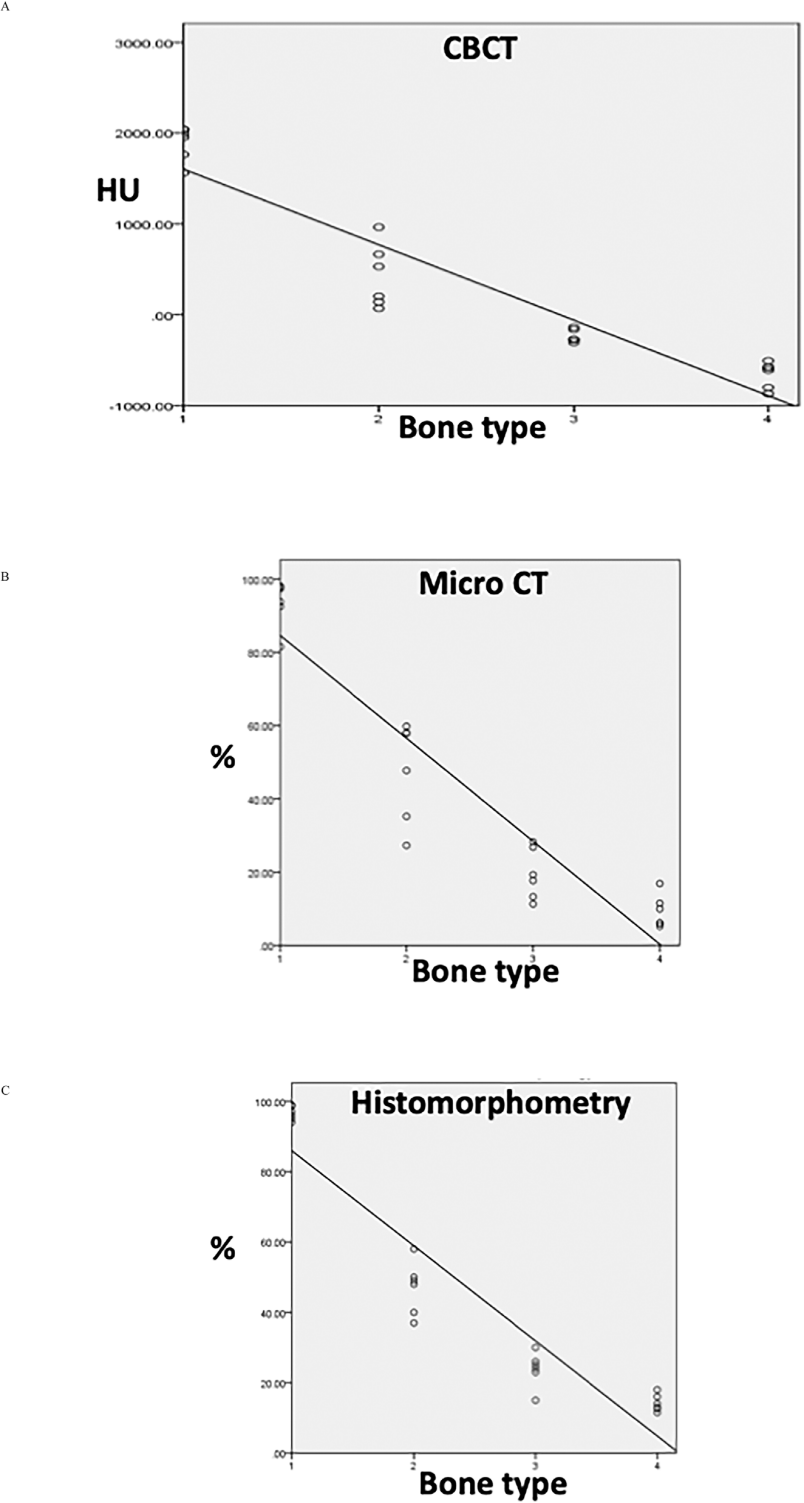
Graphic presentation of the quantitative bone type results. **A**, Presentation of the quantitative (q) cone-beam computed tomography (CBCT) data for each bone type. X-axis: bone types 1 to 4; y-axis: qCBCT data in Hounsfield units (HUs). Pearson correlation coefficient = 0.9. **B**, Quantitative micro–computed tomography data (% mineralised bone density) are represented on the y-axis. Pearson correlation coefficient = 0.9. **C**, Quantitative histomorphometric data (% mineralised bone volume density) are represented on the y-axis. Pearson correlation coefficient = 0.9. The Pearson correlation coefficients for each method are thus 0.9.

Quantitative CBCT data (in HUs) of the calibration solutions (Figure 2A) were obtained by using standard software for CBCT quantification.^23^^,^^24^ These radiographic phantom measurements of the calibration solutions with the 2 different software products (ProMax 3D and Simplant 16) yielded identical data sets, as illustrated in Figure 2B; adequate repetitions of measurements confirmed this. The quantitative measurements of the identical bone areas by CBCT (in HUs, converted to grey values), by q micro-CT and by quantitative histomorphometry were performed as illustrated pictorially by a representative example in Figure 3. The comparative analyses of the identical bone planes for each bone type by the 3 different methods (CBCT, micro-CT, histomorphometry) are illustrated in Figure 4. The data show that the Pearson correlation coefficients for each of the 3 methods used are 0.9 and thus indicate a very high degree of reproducibility by these different methodologies. The comparison between the group data and the pooling of 2 group data sets (CBCT and micro-CT; CBCT and histomorphometry [Table 1A]) revealed very strong positive Pearson correlation coefficients (*R* values of 0.97 [CBCT vs micro-CT] and 0.98 [CBCT vs histomorphometry]), with no significant difference between micro-CT and histomorphometry: *P* = .0065 (Table 1A) and coefficients of determination (*R*^2^ values) of 0.88 to 0.89 in the 3 groups (Table 1B). There was little data spread phenomena and therefore very strong curve fittings (Figures 4A–4C; Tables 1A and 1B).

**Table 1:** Comparison of bone quantification methods.

A.					
Bone quantification method		Micro CT (% bone)		CBCT (HUs)	
Micro CT (% bone**)**		———–		*R* = 0.97 (*P* < .001)	
Histomorphometry (% bone)		*P* = .065		*R* = 0.98 (*P* < .001)	

B.					
	Micro CT (% bone)		CBCT (HUs)		Histomorphometry (% bone)
Coefficient of determination (*R*^2^**)**	0.9 (*P* < .001)		0.9 (*P* < .001)		0.9 (*P* < .001)

CBCT, cone-beam computed tomography; CT, computed tomography; HUs, Hounsfield units; R, Pearson correlation coefficient.

## Discussion

For validation purposes of the qCBCT approach for bone typing, this method was compared with 2 other methods for bone typing, namely q micro-CT and quantitative histomorphometry; the exact same locations of analysis in the skeleton were used for this purpose. The data revealed that there exists a very strong correlation of the qCBCT approach with the 2 other methods (q micro-CT and quantitative histomorphometry) used for quantitative bone typing. In particular, the calibration studies with the K_2_HPO_4_ solutions and using 2 different commercially available CBCT software products revealed that identical results are generated, confirming the independence of data of different commercial software origins. Taken together, the data of these different measurements and analytical tools used for quantitative bone tissue characterisation illustrated that they are able to provide a reproducible basis for bone typing that can therefore be achieved on an observer-independent basis. These data also confirm previous findings with respect to the usefulness of CBCT in deriving HUs from grey levels^9^ and the strong linear relationship operating between these 2 parameters.^25^ Recent reviews^26^^,^^27^ indicate that the technical preconditions for such reproducible quantifications of bone structure by CBCT are indeed technically provided by more recent and thus more advanced CBCT equipment that is nowadays generally used in dental practice. It thus can be assumed that current CBCT equipment in dental clinics fulfils the requirements for the quantification of human bone types in the presurgical evaluation phase in implantology.

The double cross-checking of the CBCT bone type identification method by q micro-CT^28^ and by quantitative histomorphometry^29^ revealed Pearson correlation coefficients around 0.9 for all cross-checking computations of the 3 groups of different quantification modes (qCBCT, q micro-CT, quantitative histomorphometry) of exactly the same topographical locations in the human bone tissue. The small data spread (*R*^2^) of the pooled groups supports this view. And this again confirms the technical reliability and reproducibility of the qCBCT approach for dental practice, as well as its usefulness in assessing bone quality.^3^^,^^15^ Given this approach of a high degree of accuracy for the assurance of identical locations to be measured and quantified in this study, it is not surprising that fit of data was very strong.

However, data fit amongst the 3 quantification methods chosen for comparison and method validation purposes was not perfect, that is, the Pearson correlation coefficients were not 1.0 but were 0.9. In order to explain this finding, we need to consider the occurrence of systematic experimental errors that may have multiple sources, such as the imperfect placing of the gp markers and the ensuing slight offset relocation of the investigated bone planes of analysis in the 3 methods, which would lead to an absence of perfectly identical planes. Moreover, chemical processing methods for histology may have been associated with slight volume changes and tissue distortion effects upon dehydration; slight variabilities in the apparatus performance over time may also play a role. Furthermore, aberrations in the calibration process may contribute to such errors, suggesting that we can expect different variations to occur with each measuring method used. Such factors contribute to the limitations of the study.

In particular the high degree of accuracy obtained with the quantitative histomorphometry confirms the practical usefulness of qCBCT for the use in dental practice; however, the use of absolute values of CBCT grey values needs to be avoided in order to avoid inaccurate assessments.^30^ Moreover, the baseline histomorphometric data obtained in this study for the 4 human bone types in the male facial skeleton can serve as a baseline database pool for future studies and for the development/investigation of novel equipment in this area, and they can potentially also can serve this use in forensic medicine.

Quantitative CBCT was developed for clinical use in the dental field^10^ and also other areas of clinical medicine.^31^^,^^32^ Its practical availability in oral implantology for observer-independent identification of the local bone type at a prospective site of implant placement will be a great help to oral surgeons (both experienced and unexperienced) and to clinical researchers to provide a higher degree of reproducibility. It may also be able to improve implant outcome research through establishment of an improved homogeneity of the treatment groups. Furthermore, it is able to provide an improved time-efficiency in the clinical planning phase as it does not require additional input by colleagues. It may be able to assist experienced surgeons who are confronted with different opinions relating to the bone types present when only subjective criteria are applied, as had been suggested in various publications.^33^ Quantitative CBCT for bone typing thus has the potential to serve as a powerful tool for clinical outcome monitoring, clinical studies and prospective clinical trials to provide reproducible and objective data sets, and useful surrogate marker information.

## Conclusions

Quantitative CBCT is shown here by appropriate validation to be a valuable tool for the oral surgeon in identifying rapidly on an objective and reproducible basis the type of human bone present at prospective sites of implant placement.

## Conflict of interest

None disclosed.
